# Editorial: Advances in computer science and their impact on data acquisition and analysis in neuroscience

**DOI:** 10.3389/fncom.2024.1537106

**Published:** 2025-01-06

**Authors:** Peter Koulen, Amirfarhang Mehdizadeh, Mei-Ling Shyu, Chengcui Zhang, Shu-Ching Chen

**Affiliations:** ^1^Vision Research Center, Department of Ophthalmology, School of Medicine, University of Missouri-Kansas City, Kansas City, MO, United States; ^2^Department of Civil and Mechanical Engineering, School of Science and Engineering, University of Missouri-Kansas City, Kansas City, MO, United States; ^3^Department of Computer Science, The University of Alabama at Birmingham, Birmingham, AL, United States; ^4^Data Science and Analytics Innovation Center, University of Missouri-Kansas City, Kansas City, MO, United States

**Keywords:** artificial intelligence, biology, diagnostics, machine learning, nervous system, neuroscience, neurology, therapeutics

## Overview

Unlike other fields of biology, neuroscience and the study of the nervous system across a range of species were characterized early on by the need for the analysis of highly complex data sets. In seminal areas of neuroscience, such as electrophysiology, neuroimaging, and neurophysiology, progress in both data acquisition and data analysis was limited by the capabilities of hardware and software solutions available to researchers. At the same time, critical advances in computer science and software engineering not only led to fundamentally important new discoveries in neuroscience but were often also driven by challenges resulting from paradigm-shifting discoveries in neuroscience, such as the 1981 Nobel Prize in Physiology or Medicine. Similar synergistic relationships between computer science and neuroscience also exist in medical fields related to neuroscience, such as neurology, ophthalmology, and psychiatry, where progress in the development of novel therapeutics and diagnostics depends on the ability to more effectively acquire and analyze data, such as the 2003 Nobel Prize in Physiology or Medicine.

The seminal importance of many novel developments in computer science generates a broad impact on basic science approaches in neuroscience, often subsequently or in parallel advancing toward clinical implementation. Novel approaches in the areas of artificial intelligence and machine learning, for example, have found early sites of implementation in neuroscience and related medical fields. At the same time, developments in neuroscience, such as international collaborative efforts in connectomics and brain–computer interface research, provide new concepts and prompt new challenges for computer scientists in fundamental and applied research fields.

The Research Topic, *Advances in computer science and their impact on data acquisition and analysis in neuroscience*, brought together experts in both areas, computer science and neuroscience, with interdisciplinary teams presenting innovative approaches and synthesizing insights toward the synergistic advancement of their respective fields. The Research Topic covers clinically and scientifically highly relevant topics ranging from disease diagnostics to the prognosis of outcomes after therapeutic intervention and the development of neural networks for image recognition and the identification of structures of the nervous system. It encompasses two *Original Research* articles, two *Methods* articles, and one *Brief Research Report*.

Research discussing how data distribution characteristics can be utilized to capture biomarkers associated with Alzheimer disease subtypes is presented by Smith and Climer. Specifically, the study targets the combined complexities of the multiple subtypes and of the diagnostic biomarkers of late-onset form of Alzheimer disease. The former is characterized by ranges of disparate risk factors, pathological manifestations, and clinical traits, and the latter is by different methods used for quantitation and statistical evaluation, resulting in insufficient evaluation metrics. Smith and Climer elegantly introduce and validate a novel conceptual framework with an evaluation metric based on the distribution of diagnostic biomarker values, rather than on the magnitude of the values. The study further provides evidence for the effectiveness of its strategy when identifying subtypes of heterogeneous diseases, such as the late-onset form of Alzheimer disease.

Targeting the same complex disease spectrum, Alzheimer disease, and the same scientific problem, disease classification, Ferrante et al. describe their novel approach of converting a standard neural network into a Bayesian neural network with an estimation of the variability of predictions. Specifically, the authors identify uncertainty estimation as a key factor for the implementation of deep learning in clinical settings, with their approach producing improved trust in and usability of automatic systems, resulting in improved performance in disease classification tasks (Ferrante et al.).

Qiu et al. also target classification tasks but focus on a more fundamental problem, namely the identification and classification of neurons, synapses, and other structural features of the nervous system. With the goal of facilitating a deeper understanding of the nervous system, the authors target the complexities of three-dimensional point cloud data sets, where classification and information extraction efforts are hampered by noise, sparsity, and disorder of the data. Performing both qualitative and quantitative experiments, the authors provide evidence for the efficacy of their approach for the sampling classification task as a novel tool for neuroscientists and neuroanatomists (Qiu et al.).

Cao et al. provide a novel approach as an alternative to current graph neural network frameworks in the area of image recognition. By using a linear graph neural network framework, the authors developed a high-order neighbor propagation method to capture and learn the representation information of high-order neighbor nodes without using a multi-channel architecture and depth map neural network. Further, by employing a multi-scale feature fusion mechanism, which comprehensively considers the propagation information of different orders, the authors are able to provide experimental data showing the superiority of their model to existing methods currently in use (Cao et al.).

Tong et al. focus on surgical endovascular clot removal and mechanical thrombectomy for the treatment of acute ischemic stroke. Specifically, the authors address the clinical problem that this treatment approach, even with successful reperfusion, yields a favorable outcome only in a subset of acute ischemic stroke patients with large vessel occlusion. They explore the use of machine learning, to generate a model for predicting health outcomes after mechanical thrombectomy, accompanied by methods to enhance the interpretability of the model (Tong et al.). Tong et al. were able to identify an effective approach for predicting the prognosis of acute ischemic stroke patients with large vessel occlusion especially for patients outside the standard 6 h therapeutic window, which might facilitate future clinical decision-making using their predictive model.

In summary, the Frontiers Research Topic on *Advances in computer science and their impact on data acquisition and analysis in neuroscience*, provides novel contributions exemplifying how interdisciplinary work in computer science and neuroscience can synergistically improve data analysis in a wide range of scientific and medical utilities increasing both scientific knowledge and the value of tools in clinical diagnostics, prognostics, and therapeutics, altogether reducing the socio-economic burden of disease conditions affecting the nervous system.

